# Ethnology: Crania Britannica

**Published:** 1853

**Authors:** John M. Galt

**Affiliations:** Superintendent and Physician of the E. S. Asylum of Virginia


ARTICLE II.—*Ethnology:—Crania Britannica.*—By JOHN M. GALT, M. D., Superintendent and Physician of the E. S. Asylum of Virginia.

One of the most distinguished philosophers ever produced by the English race, has made an assertion which has now become sufficiently hackneyed; but this should not blind us to the real merit of the well-known quotation from Pope, that—"the proper study of mankind is man." Every fact, indeed, bearing upon either the physical or the psychological manifestations of the human race, as concerning ourselves, remotely or directly, is worthy of study and attention. Hence it is that some of the greatest thinkers, such as Dr. Prichard in England, and Dr. Morton in America, have devoted, with extreme ardour, long and active lives to the elucidation of this branch of human inquiry. And the honour and glory which each of these great men has conferred by his labours, on the land of his nativity, shows how universally their exertions have been appreciated. There is no need or room, however, for further preliminary remarks of this character; an undertaking of the kind should possess a sufficient recommendation to all physicians, if the result alike of research and of talent. And we proceed, therefore, to offer a few particulars to the medical world concerning the contemplated publication.

The work, then, whose title we transcribe further on, is intended to supply the deficiency that at present exists in relation to the remains of the ancient inhabitants of Great Britain, obtained from barrows and other tombs, and has a motto prefixed, from "Wilson's Archeology of Scotland," which appeals to Americans almost as much as to the citizens of Great Britain, being to this effect—"They are our ancestry, even though we may question our lineal descent; our precursors, if not our progenitors. From them we derive our inheritance and birth-right; nor, amid all the later minglings of races, can we assume that no drop of their blood mingles in our veins." We hope, indeed, as the work is to be of a costly description, that it will be procured for libraries generally, public and private, in the United States. Men of science in all parts of the globe, form a vast republic, who should mutually aid and assist each other in bearing forward the one great cause in which they are so zealously engaged.

The circular of the authors contains a full account of the nature of the work—Proposals for publishing an important national work, illustrative of the Ethnology of Great Britain and Ireland—Crania Britannica, or Delineations of the Skulls of the Aboriginal Inhabitants of the British Islands, and of the Races immediately succeeding them; together with notices of their other Remains. By Joseph Barnard Davis, F. S. A., member of the Royal College of Surgeons of England; of the Archeological Institute of Great Britain and Ireland; Associate of the Ethnological Society, and of the British Archeological Association; and John Thurman, M. D., F. S. A.; Licentiate of the Royal College of Physicians of London; Member of the Archeological Institute of Great Britain and Ireland; of the British Archeological Association; and of the Ethnological Society of London; Author of Papers on the Ethnology and Archeology of England.

The qualifications of these gentlemen are such as to enable us to vouch for the excellence of the forthcoming effort: We doubt not that it will be every thing that it should be—constituting a most able and valuable contribution to the science of Ethnography. The cost of the work, which will come out in six decades, will be thirty dollars; or one guinea for each decade (published quarterly) to be paid on delivery. It is to be "privately printed," issued in London, and strictly confined to subscribers, whose names are requested to be communicated to the Authors only, that its further progress may be insured, for it is distinctly stated by them, that it is proposed to publish the series of decades, "if a sufficient number of subscribers can be obtained to prevent pecuniary loss." Each of the six numbers is intended to contain ten lithographic plates, in imperial quarto, with descriptive letter press, giving the history of the exhumation of each cranium, an account of the antiquities disinterred with it, accompanied by figures, &c., together with exact admeasurements. Some of the most complete and varied collections in the kingdom of Great Britain, have been placed at the entire disposal of these writers, one of whom, (Dr. Thurman) some time since had a grant from the Committee of Recommendations of the Royal Society for prosecuting an inquiry on this subject, with the results of which it is hoped to enrich the work under consideration. Neither pains nor expense will be spared in accumulating further materials from every part of the British Isles, in procuring the best artistic skill for their delineation, and in producing such a work as befits the real importance of the science which it is designed to illustrate.

Mr. Davis writes us word, that subscriptions, the names of subscribers, &c., may be sent either to himself, directed —Joseph Barnard Davis, Esq., Shelton, Staffordshire, England; or to Franklin Peale, Esq., United States Mint, Philadelphia, Pennsylvania.

Amongst those who have already subscribed to this publication, may be mentioned the following distinguished names; there being also a number of the crowned heads, and of the highest nobility in Transatlantic countries—Professor L. Agassiz, Cambridge, Massachusetts; Robert Stephenson, Esq., M. P., F. R. S.; Hon. E. G. Squier, F. S. A., New York; Professor G. Vrolic, Amsterdam, Holland; Professor E. G. Carns, M. D., Dresden, Saxony, (Physician to the king of Saxony); George Combe, Esq., Edinburg, Scotland; Chevalier Bunsen, Royal Library, Berlin; John Bowring, L. L. D., England; Col. C. C. Biddle, Philadelphia; W. Farr, Esq., M. D., Somerset House, England; Professor J. Van der Hover, Leyden; Professor D. Wilson, L. L. D., Toronto, Canada West. Amonst the monarchs subscribing may be mentioned the King of the Belgians, the King of Denmark, the King of Sweden and Norway, &c. And of the nobility, the Duke of Buccleugh, the Duke of Newcastle, the Rt. Hon. Lord Londesborough, F. R. S., &c. JOHN W. GALT.

Jan. 30, 1854.

